# CNTNAP2: isoform- and context-specific functions in neurological disorders and cancer

**DOI:** 10.3389/fncel.2026.1803486

**Published:** 2026-04-24

**Authors:** Yu Ye, Lin Zhu, Danling Wang

**Affiliations:** 1 School of Basic Medical Sciences, Hengyang Medical School, Institute of Cytology and Genetics, University of South China, Hengyang, Hunan, China; 2 Hengyang Medical School, University of South China, Hengyang, Hunan, China; 3 Institute for Future Sciences, University of South China, Changsha, Hunan, China; 4 MOE Key Lab of Rare Pediatric Diseases, School of Life Sciences, University of South China, Changsha, Hunan, China

**Keywords:** autoimmune encephalitis, cancer, CASPR2, CNTNAP2, *CNTNAP2-201*, *CNTNAP2-203*, neurodevelopmental disorder, psychiatric disorder

## Abstract

Contactin-associated protein-like 2 (*CNTNAP2*) is one of the largest and most evolutionarily conserved genes in the human genome that increasingly recognized as a pleiotropic and context-dependent regulator of human disorders. Genetic, immunological, and transcriptomic studies have implicated *CNTNAP2* in a broad spectrum of neurological and psychiatric disorders, autoimmune encephalitis, and cancer. Early work focused primarily on the full-length isoform *CNTNAP2-201*, which encodes CASPR2 or CNTNAP2, and plays essential roles in neuronal development, axon-glia interactions, synaptic transmission, interneuron maturation, and maintenance of excitatory-inhibitory balance. Disruption of these functions contributes to impaired cortical connectivity and network dysfunction in neurodevelopmental disorders. Recent discoveries have substantially expanded this view by revealing isoform-specific and proteolytic fragment-dependent functions of CNTNAP2. Proteolytic processing of CNTNAP2 generates bioactive extracellular and intracellular fragments that regulate calcium homeostasis, gene expression, and neuronal network activity. In parallel, the short isoform *CNTNAP2-203* has recently emerged as an oncogenic driver in oral squamous cell carcinoma, where its selective upregulation amplifies EGFR-E2F1 signaling and promotes tumor progression. This review synthesizes current knowledge of CNTNAP2 biology, highlighting isoform- and context-specific mechanisms and outlining key unanswered questions relevant to both neurological disease and cancer.

## Introduction

Contactin-associated protein-like 2 (*CNTNAP2*) is one of the largest and most evolutionarily conserved genes in the human genome (Rodenas-Cuadrado et al., 2013). Two validated transcripts—*CNTNAP2-201*, the canonical full-length isoform, and *CNTNAP2-203*, a shorter isoform—are expressed across a range of human tissues, including the central nervous system (CNS), peripheral nervous system (PNS), and multiple tumor types (St George-Hyslop et al., 2023; Zhu et al., 2025).

Genetic disruption of *CNTNAP2* has been robustly implicated in a wide spectrum of neurodevelopmental disorders, including autism spectrum disorder (ASD), intellectual disability (ID), and specific language impairment (SLI) (El-Cheikh Mohamad et al., 2023; Peñagarikano et al., 2011; Wang and Feldman, 2024). In addition, CNTNAP2 variants have been associated with epilepsy, schizophrenia, Tourette syndrome, and attention deficit/hyperactivity disorder (ADHD) (Elia et al., 2010; Friedman et al., 2008; Verkerk et al., 2003). Beyond inherited variation, autoantibodies targeting CNTNAP2 have been linked to autoimmune limbic encephalitis, further underscoring its relevance in acquired neurological disease (Saint-Martin et al., 2019).

Recent evidence indicates that CNTNAP2 dysregulation is not confined to the nervous system. Altered CNTNAP2 expression has been reported in several malignancies, including glioblastoma, oligodendroglioma, laryngeal squamous cell carcinoma (LSCC), and oral squamous cell carcinoma (OSCC) (Bralten et al., 2010; Li et al., 2018; Parris et al., 2014; Rautajoki et al., 2022; Thorell et al., 2009; Zhang J. et al., 2024). These alterations may result from diverse mechanisms, such as chromosomal rearrangement, somatic mutation, and isoform-specific dysregulation (Bralten et al., 2010; Wang and Feldman, 2023).

Collectively, these findings position CNTNAP2 as a pleiotropic regulator implicated in both neurological and oncological pathologies. In this review, we synthesize current knowledge of CNTNAP2 biology, with a particular emphasis on isoform- and variant-specific functions, highlighting established molecular mechanisms as well as emerging concepts that may help clarify its context-dependent roles in health and disease.

## *CNTNAP2* gene isoforms and protein variants

**FIGURE 1:**
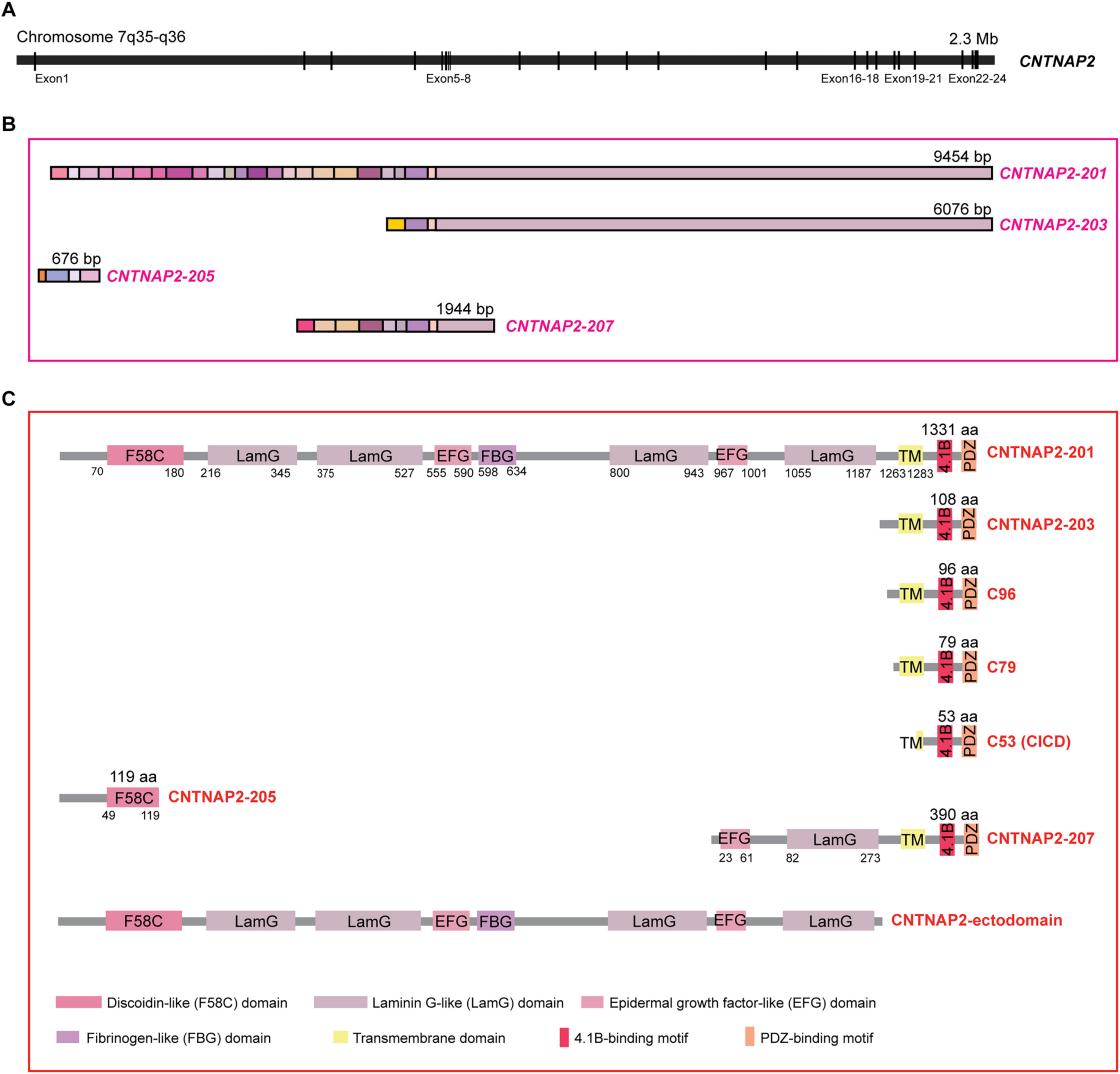
Genomic organization and protein structure of contactin-associated protein-like 2 (CNTNAP2). **(A)** The *CNTNAP2* gene is located on chromosome 7q35, spanning ∼2.3 million base pairs and having 24 exons. **(B)** Schematic of the four protein-coding *CNTNAP2* transcripts. Colored rectangular boxes indicate individual exons. **(C)** Schematic illustration of the CNTNAP2 protein and its derived fragments. Distinct functional domains are shown as colored rectangles, with domain names and abbreviations indicated in the legend.

*CNTNAP2* is located on chromosome 7q35, spanning ∼2.3 million base pairs (bp) and accounting for ∼1.5% of the chromosome 7 (Nakabayashi and Scherer, 2001). Its full-length, canonical open reading frame contains 24 exons (Rodenas-Cuadrado et al., 2014). According to Ensembl (release 114, May 2025), the *CNTNAP2* locus gives rise to four protein-coding transcripts: the full-length *CNTNAP2-201* (9,454 bp, 24 exons), *CNTNAP2-203* (6,076 bp, 4 exons), *CNTNAP2-205* (676 bp, 4 exons), and *CNTNAP2-207* (1,944 bp, 9 exons, Figure 1).

**FIGURE 2:**
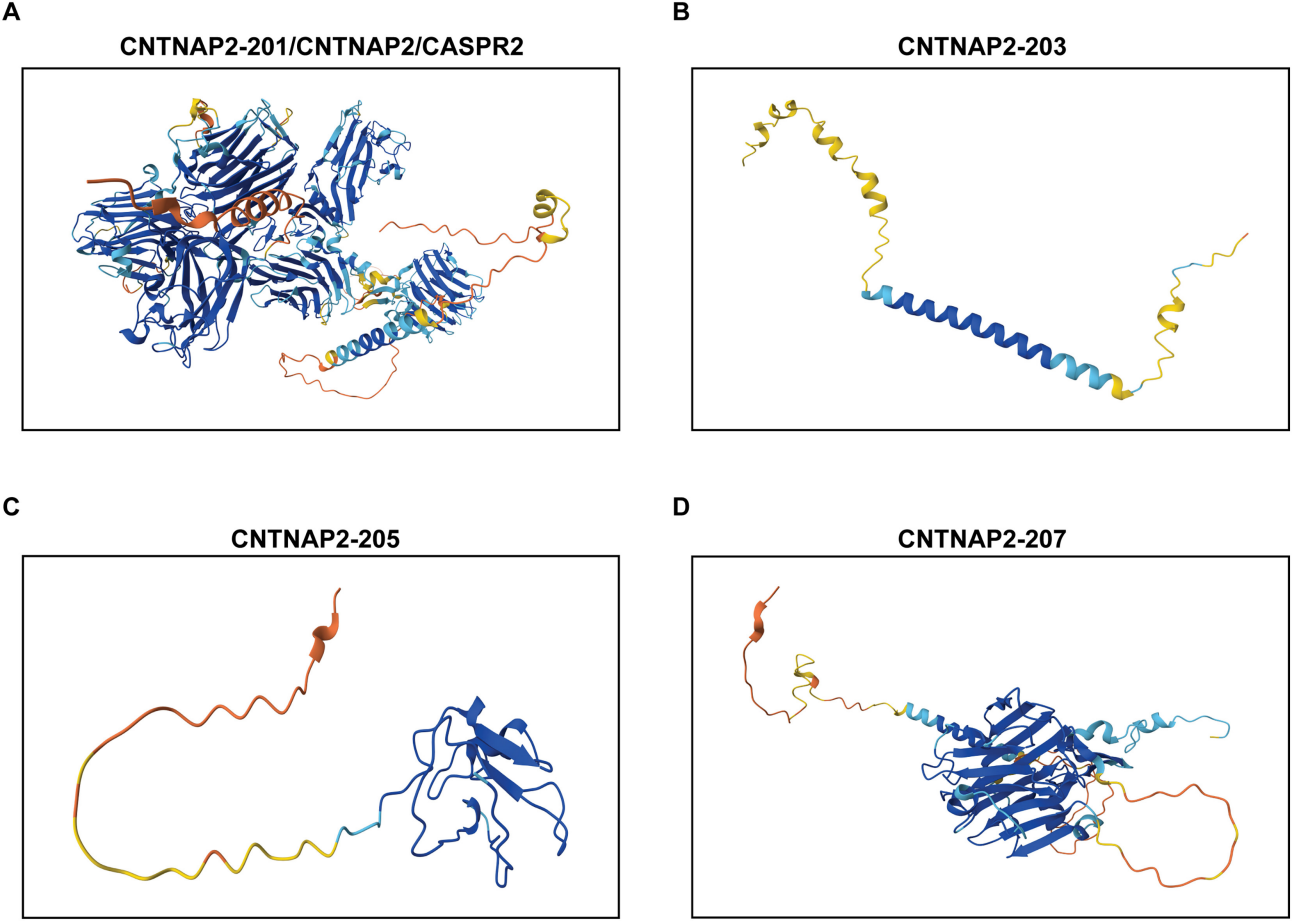
Predicted three-dimensional structure of the contactin-associated protein-like 2 (CNTNAP2) isoforms. Predicted structures of CNTNAP2 isoforms CNTNAP2-201 **(A)**, CNTNAP2-203 **(B)**, CNTNAP2-205 **(C)**, and CNTNAP2-207 **(D)** were generated using AlphaFold. Model confidence for each residue is represented by the predicted local distance difference test (pLDDT) score and color-coded as follows: blue (pLDDT > 90), very high confidence; light bule (90 > pLDDT > 70), high confidence; yellow (70 > pLDDT > 50), low confidence; and orange (pLDDT < 50), very low confidence.

The full-length isoform *CNTNAP2-201* encodes contactin-associated protein-like 2 (CASPR2), also called CNTNAP2 or CNTNAP2-201, a single-pass transmembrane glycoprotein composed of 1,331 amino acids. CASPR2 features a large extracellular region composed of one N-terminal discoidin-like (F58C) domain, four laminin G-like (LamG) domains, two epidermal growth factor (EGF)-like domains, and one fibrinogen-like domain (Figure 1C; Poot, 2017). Its short intracellular tail includes a 4.1B-binding motif and a type II PDZ-binding motif (Figure 1C; Zou et al., 2017). This modular architecture forms a globular, F-shaped structure organized into three lobes, with intrinsic flexibility to facilitate the interactions with multiple binding partners and regulate subcellular localization in neurons (Figure 2A; Lu et al., 2016).

The *CNTNAP2-203* transcript maps to the 3’ end of the CNTNAP2 locus and encodes a predicted ∼12-kDa protein of 108 amino acids, corresponding to residues 1,224–1,332 of CASPR2 (Figure 2B). This isoform retains the transmembrane and intracellular domains of CASPR2 but lacks the majority of the extracellular region (Figure 1C). *CNTNAP2-207* is predicted to encode a 390-amino-acid protein containing a small extracellular segment with one LamG and one EGF-like domain, followed by transmembrane/intracellular domains identical to those of CASPR2 and CNTNAP2-203 (Figures 1C, 2C). In contrast, *CNTNAP2-205* encodes a truncated 119-amino-acid protein corresponding to the N-terminal sequence of the first two exons of CASPR2 (Figures 1C, 2D).

Among these transcripts, *CNTNAP2-201* and *CNTNAP2-203* are the only isoforms with confirmed RNA and protein expression across human tissues (Chen et al., 2015; Traka et al., 2003). Accordingly, this review focuses on these two isoforms, with particular emphasis on their expression pattern, functional diversity, and relevance to human disease.

## *CNTNAP2* and human disorders

Dysregulation of CNTNAP2 has been increasingly linked to a broad spectrum of human diseases, including neurodevelopmental and psychiatric disorders, autoimmune encephalitis, and cancer (Table 1). These diverse associations highlight CNTNAP2 as a pleiotropic, context-dependent regulator of human health and disease (Bakkaloglu et al., 2008; Liao et al., 2024; Peñagarikano and Geschwind, 2012; Zhu et al., 2025).

**TABLE 1:** Human diseases associated with contactin-associated protein-like 2 (CNTNAP2) dysregulation.

Category	Disease/condition	Type of dysregulation	Functional consequence	Representative references
Neurodevelopmental and psychiatric disorders	Autism spectrum disorder	Rare mutations, risk variants, altered expression	Impaired neuronal connectivity, synaptic dysfunction, altered social behavior	Belloso et al., 2007; Alarcón et al., 2008; Bakkaloglu et al., 2008; Arking et al., 2008; Jackman et al., 2009; O’Roak et al., 2011; Anney et al., 2012; Rodenas-Cuadrado et al., 2014; Carraro et al., 2019; Zhang Q. et al., 2024
	Intellectual disability, language impairment	Loss-of-function mutations, deletions	Severe speech delay, cognitive impairment, abnormal cortical development	Zweier et al., 2009; Vernes et al., 2008; Mikhail et al., 2011; Gregor et al., 2011; Poot et al., 2010; Newbury et al., 2011; Whitehouse et al., 2012
	Epilepsy/epileptic encephalopathy	Homozygous/compound heterozygous mutations, deletions	Cortical dysplasia, seizures, hyperexcitability	Strauss et al., 2006; Mefford et al., 2010
	Schizophrenia, tourette syndrome, Pitt-Hopkins syndrome, attention deficit/hyperactivity disorder	Genome-wide association studies risk alleles, copy number variant	Disrupted neuronal circuits, psychiatric vulnerability	Verkerk et al., 2003; Zweier et al., 2007; Petrin et al., 2010; Elia et al., 2010; Peñagarikano and Geschwind, 2012
Autoimmune and neurological disorders	Autoimmune limbic encephalitis, Morvan syndrome	Autoantibodies targeting CASPR2 protein	Neuroinflammation, seizures, psychiatric symptoms, neuromyotonia	Lancaster et al., 2011; Irani and Vincent, 2012; Melzer et al., 2012; Faivre-Sarrailh and Devaux, 2013; Joubert et al., 2016b; Patterson et al., 2018; Fernandes et al., 2019; Saint-Martin et al., 2019; Muñiz-Castrillo et al., 2020; Ramanathan et al., 2021; Gendre et al., 2021; Comperat et al., 2022
Cancer	Childhood neuroblastoma, laryngeal squamous cell carcinoma, hypopharyngeal squamous cell carcinoma	Aberrant CNTNAP2 expression	Unfavorable tumor, chemosensitive tumor, concurrent esophageal squamous cell carcinoma	Thorell et al., 2009; Li et al., 2018; Zhang J. et al., 2024
	Oligodendroglioma	Gene rearrangement	Recurrent tumor	Rautajoki et al., 2022
	Oral squamous cell carcinoma	Isoform upregulation (CNTNAP2-203)	Promotes proliferation, poor prognosis	Zhu et al., 2025

### Implications of CNTNAP2 in neurodevelopmental and psychiatric disorders

Genetic alterations in *CNTNAP2* have been reported in multiple neurodevelopmental disorders (NDDs) and psychiatric conditions, including ASD, ID, SLI, epilepsy, schizophrenia, obsessive compulsive disorder, Gilles de la Tourette syndrome (GTS), cortical dysplasia-focal epilepsy (CDFE), bipolar disorder, and dyslexia (D’Onofrio et al., 2023; El-Cheikh Mohamad et al., 2023; Ji et al., 2013; Peñagarikano et al., 2011; Strauss et al., 2006; Verkerk et al., 2003; Wang and Feldman, 2024).

The first genetic link between *CNTNAP2* and NDDs was reported in 2003, when a family with GTS was found to carry a chromosomal insertion at 7q35–36, which disrupts intron 8 of *CNTNAP2* (Verkerk et al., 2003). Subsequent studies identified additional chromosomal rearrangements involving *CNTNAP2* in ASD (Burbach and van der Zwaag, 2009), SLI (Poot et al., 2010), epilepsy, and schizophrenia (Friedman et al., 2008). Copy number variant analyses further revealed *de novo* heterozygous deletions within *CNTNAP2* in individuals with Pitt-Hopkins syndrome (PTHS) (Blake et al., 2010), ASD (Alarcón et al., 2008), ADHD (Elia et al., 2010), stuttering (Petrin et al., 2010), and ID (Mikhail et al., 2011), among others. Collectively, these observations support CNTNAP2 haploinsufficiency as a key pathogenic mechanism underlying a spectrum of NDDs and psychiatric phenotypes.

In addition to structural variants, point mutations and microdeletions affecting *CNTNAP2* have also been reported (Al-Murrani et al., 2012; Baig et al., 2017; Bakkaloglu et al., 2008; Gregor et al., 2011; Jackman et al., 2009; Mikhail et al., 2011; O’Roak et al., 2011; Petrin et al., 2010; Strauss et al., 2006). Notably, many heterozygous missense variants are inherited from unaffected parents, raising important questions regarding their penetrance and pathogenicity. A particularly compelling causal association was identified in an Old Order Amish population, in which 13 probands carried a homozygous single-base deletion at nucleotide 3,709 in exon 22. This mutation introduces a premature stop codon (I1253X), predicted to produce a truncated protein lacking the transmembrane and intracellular domains (Jackman et al., 2009; Strauss et al., 2006). Functional studies demonstrated that CNTNAP2-I1253X is secreted into the extracellular space, resulting in loss of membrane anchoring and likely disruption of intercellular adhesion signaling (Falivelli et al., 2012). In induced pluripotent stem cells (iPSC)-derived models from affected individuals, forebrain organoids exhibit increased volume and total cell number, and correction of the mutation using CRISPR-Cas9 largely rescues this phenotype (de Jong et al., 2021). Clinically, individuals harboring this mutation present with a complex syndrome characterized by CDFE, delayed motor milestones, seizures, language regression, and behavioral abnormalities, including ADHD and autism (Jackman et al., 2009).

Genome-wide association studies (GWAS) have further implicated *CNTNAP2* in complex neurological disorders, including language impairment, autism, dyslexia, schizophrenia, and depression (Alarcón et al., 2008; Anney et al., 2012; Arking et al., 2008; Elia et al., 2010; Hongyao et al., 2023; Ji et al., 2013; Newbury et al., 2011; Nord et al., 2011; Peter et al., 2011; Uddin et al., 2021; Vernes et al., 2008; Zhang et al., 2025). Notably, SLI has been strongly associated with a cluster of single-nucleotide polymorphisms (SNPs) within intron 13–14 (Newbury et al., 2011; Vernes et al., 2008). The SNP rs2710102 has been repeatedly linked to ASD (Alarcón et al., 2008; Newbury et al., 2011; Shiota et al., 2021) and, together with rs759178, rs17236239, and rs2538976, has also been associated with early communicative behaviors in unaffected individuals (Newbury et al., 2011). Another variant, rs7794745 in intron 2, has likewise been associated with increased autism risk (Arking et al., 2008).

**FIGURE 3:**
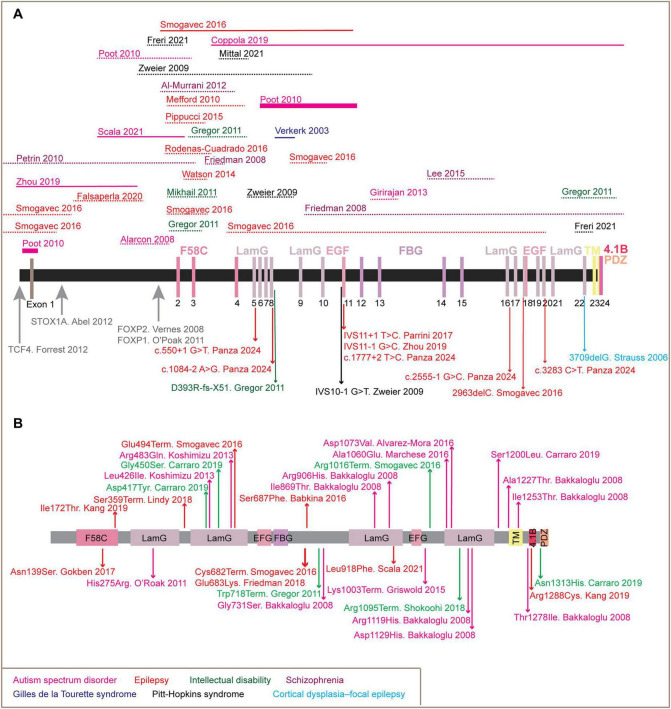
Schematic overview of disease-associated mutations in the contactin-associated protein-like 2 (CNTNAP2). **(A)** Genomic organization of the *CNTNAP2* gene, showing its 24 exons and the protein domains they encode. Dash lines above the gene indicate gross deletion, while solid lines represent gross insertions or duplications. Thick solid lines represent chromosomal translocations involving *CNTNAP2*. Arrows below the gene denote small deletions/insertions or splicing mutations. Large gray arrows mark experimentally identified binding regions for the transcription factors: TCF4, STOX1A, FOXP1, and FOXP2. **(B)** Missense or non-sense mutations in the CNTNAP2 protein sequence are marked by arrows. Each mutation is annotated with the corresponding reference (author names and publication year), and associated disease phenotypes are color-coded as indicated in the legend. Domain abbreviations are as follows: F58C, discoidin-like domain; LamG, laminin G-like domain; EFG, epidermal growth factor-like domain; FBG, fibrinogen-like domain; TM, transmembrane domain; 4.1B, protein 4.1B-binding motif; and PDZ, PDZ-binding motif.

According to the Human Gene Mutation Database (HGMD, accessed Oct 2025^1^), *CNTNAP2* variants associated with NDDs and psychiatric disorders include 29 missense/non-sense mutations, three small deletions/insertions, 27 gross deletions, eight splicing mutations, and seven chromosomal rearrangements (including gross insertions, duplication, and complex rearrangement) (Figure 3 and Table 1). Notably, the majority of pathogenic variants map to the extracellular domain, suggesting that the full-length *CNTNAP2-201*, rather than the shorter *CNTNAP2-203*, is primarily responsible for CNTNAP2-associated CNS phenotypes.

## *CNTNAP2* and autoimmune disorders

Autoantibodies directed against CASPR2, the full-length protein encoded by *CNTNAP2-201*, define a group of autoimmune neurological conditions that include autoimmune encephalitis, limbic encephalitis (LE), and Morvan’s syndrome (Table 1). These conditions are clinically heterogeneous and may present with various symptoms such as neuromyotonia, neuropathic pain, cerebella ataxia, epilepsy, insomnia, confusion, etc., (Becker et al., 2012; Faivre-Sarrailh and Devaux, 2013; Irani and Vincent, 2012; Irani et al., 2010; Joubert et al., 2016b,^2017^; Lancaster et al., 2011; Melzer et al., 2012; Muñiz-Castrillo et al., 2020).

In CASPR2-associated LE, inflammation primarily affects the medial temporal lobes of the limbic system. Patient-derived antibodies target the extracellular epitopes of CASPR2 (Olsen et al., 2015; Pinatel et al., 2015), leading to antibody-mediated internalization of the protein and disruption of its interactions with key molecular partners. These processes impair CASPR2 trafficking and downstream cellular functions (Fernandes et al., 2019; Patterson et al., 2018; Saint-Martin et al., 2019). The presence of CASPR2 autoantibodies in cerebrospinal fluid, together with the high expression of *CNTNAP2* in the hippocampus and other limbic regions, supports a direct pathogenic role for these CASPR2 autoantibodies within the CNS.

CASPR2 antibodies have also been implicated in PNS pathology. In patients with neuropathic pain, CASPR2-specific antibodies were detected in 76 out of 147 cases, with pain symptoms predominantly affecting the limbs (Ramanathan et al., 2021). Similar findings have been reported in patients with paresthesia, in whom CASPR2 autoantibodies reacted with sensory C-fibers of peripheral nerves (Gendre et al., 2021). In autoimmune neuromyotonia, CASPR2 antibodies frequently coexist with peripheral nerve hyperexcitability, manifesting as muscle spasms, tremor, and muscle atrophy (Comperat et al., 2022). Together, these findings indicate that CASPR2 dysfunction contributes to both central and peripheral autoimmune neurological symptoms.

Epitope mapping studies have demonstrated that CASPR2 autoantibodies, typically composed of mixed IgG1 and IgG4 isotypes, consistently target extracellular epitopes, regardless of the clinical presentation (Joubert et al., 2016a). To date, no intracellular epitopes have been identified with CASPR2 autoantibodies, reinforcing the concept that CASPR2-associated autoimmune disorders primarily arise from pathogenic targeting of protein regions encoded by *CNTNAP2-201*.

### Implications of CNTNAP2 in cancer

Emerging evidence indicates *CNTNAP2* dysregulation contributes to tumor progression and may hold prognostic value across multiple malignancies (Table 1). Several studies suggest a tumor-suppressive role for CNTNAP2. In childhood neuroblastoma, *CNTNAP2* transcript levels are significantly lower in unfavorable compared with favorable tumors (Thorell et al., 2009). Similarly, recurrent oligodendrogliomas display reduced *CNTNAP2* mRNA levels relative to primary tumors (Rautajoki et al., 2022), with gene rearrangement-driven downregulation correlating with higher tumor grade and poorer prognosis. In LSCC, *CNTNAP2* expression is reduced in chemosensitive compared with chemoresistant tumors, suggesting its potential as a predictive biomarker for treatment response (Li et al., 2018). Decreased *CNTNAP2* transcript levels have been observed in hypopharyngeal squamous cell carcinoma (HPSCC) with concurrent esophageal squamous cell carcinoma (ESCC), compared to pure HPSCC (Zhang J. et al., 2024). Collectively, these observations support a tumor-suppressive function of CNTNAP2 in multiple cancer types, although the contribution of individual isoforms and the underlying molecular mechanisms remain largely unexplored.

Notably, recent findings also suggest a potential oncogenic role of a specific *CNTNAP2* isoform. In OSCC, *CNTNAP2-203*, but not *CNTNAP2-201*, is preferentially expressed and upregulated in malignant epithelial cells. Elevated *CNTNAP2-*203 expression correlates with poorer patient prognosis, implicating this short isoform as a pathological driver in cancer biology.

## *CNTNAP2* gene expression and its regulation

### Expression pattern of CNTNAP2-201 in the nervous system

Most studies of *CNTNAP2* expression have focused on the full-length isoform *CNTNAP2-201*, which exhibits tightly regulated temporal and spatial expression patterns in the human brain and spinal cord.

In the adult nervous of system, *CNTNAP2-201* expression was first characterized by Poliak et al. (1999) using Northern blotting with a *CNTNAP2-201*-specific probe [EST R13972, 286–2725 bp, (Poliak et al., 1999)]. Predominant expression of *CNTNAP2-201* was observed in both the brain and spinal cord. Within cortical and limbic regions—including the occipital, frontal, and temporal lobes, amygdala, and hippocampus—two transcripts (∼9 and ∼10 kb) were detected at comparable levels. In contrast, subcortical regions such as medulla, putamen, caudate nucleus, corpus callosum (CC), substantia nigra, subthalamic nucleus, thalamus, and spinal cord primarily express the ∼9 kb transcript (Poliak et al., 1999). Given that the probe was specific to *CNTNAP2-201*, these findings suggest regional differences in post-transcriptional regulation or alternative splicing. Consistently, *in situ* hybridization using *CNTNAP2-201* probes (NM_014141.3, 1,343–2,496 bp) demonstrated enrichment in cortical layer II–IV of the frontal and temporal cortex (Bakkaloglu et al., 2008).

During fetal development, *CNTNAP2-201* expression is enriched in the frontal and anterior temporal lobes, striatum, and dorsal thalamus, mirroring the cortico-striato-thalamic circuitry that supports higher cognitive functions such as language and behavior control (Abrahams et al., 2007). This spatial distribution is consistent with clinical observations linking *CNTNAP2* (predominantly *CNTNAP2-201*) to neurodevelopmental disorders characterized by ID, autism traits, and language impairment (D’Onofrio et al., 2023).

Additional evidence supports the notion that CNTNAP2 expression and function in language-related circuitry is a conserved convergence point across species. In zebra finches, *Cntnap2* mRNA is highly expressed in the robust nucleus of the arcopallium and the lateral magnocellular nucleus of the nidopallium in adult males that learn to sing, whereas no such enrichment is observed in females that do not undergo vocal learning (Panaitof et al., 2010). This sexually dimorphic expression pattern parallels the enrichment of CNTNAP2 in language-associated regions of the human brain. Subsequent studies have shown that Cntnap2 protein localizes to projection neurons and their long-range axonal terminals within the song nuclei (Condro and White, 2014), suggesting a role in the establishment and maintenance of microcircuitry and long-range connectivity underlying vocal communication.

Despite strong evolutionary conservation (∼94% amino acid identity between human and mouse, and ∼99.5% between human and chimpanzee), marked interspecies differences in cortical *CNTNAP2* expression have been observed (Abrahams et al., 2007; Alarcón et al., 2008; Panaitof et al., 2010). In contrast to the pronounced frontal cortical enrichment seen in humans, rodents display relatively limited cortical expression, with highest *Cntnap2* levels in posterior brain regions such as the olfactory bulb, ventricular zones, striatum, and thalamus (Abrahams et al., 2007). These differences suggest that *CNTNAP2* may have contributed to cortical evolution and human-specific cognitive specialization (Rodenas-Cuadrado et al., 2014).

The temporal dynamics of *CNTNAP2-201* expression during cortical development have been further elucidated using iPSCs-based differentiation models. St George-Hyslop et al. (2023) showed that *CNTNAP2-201* expression is absent in undifferentiated pluripotent stem cells, expressed at low levels in cortical progenitors (day 30), and strongly induced in postmitotic neurons (days 50–80). These *in vitro* observations are consistent with *in vivo* transcriptomic data from the developing human frontal cortex Burke et al. (2020)^2^, in which *CNTNAP2-201* expression becomes detectable by postconception week 8 and peaks around week 12, corresponding to the stage of postmitotic neuronal maturation (St George-Hyslop et al., 2023).

### Expression pattern of CNTNAP2-203 in the nervous system

Compared with the canonical *CNTNAP2-201* isoform, far fewer studies have examined the expression dynamics of other *CNTNAP2* transcripts. *CNTNAP2-203* is detectable in the human frontal cortex as early as postconception week 8, with expression levels increasing until postconception week 12, paralleling early cortical development (St George-Hyslop et al., 2023).

However, iPSC-based cortical differentiation models reveal a distinct temporal pattern for *CNTNAP2-203* relative to *CNTNAP2-201. CNTNAP2-203* displays higher overall expression levels and remains relatively constant across developmental stages, from pre-differentiated pluripotent stem cells to neuronal progenitors and postmitotic neurons (St George-Hyslop et al., 2023). This contrasts with the stage-specific induction of *CNTNAP2-201* and points to unresolved questions about the regulatory mechanisms governing *CNTNAP2-203* expression during neural development. In the same study, *CNTNAP2-207* expression was also examined but remained undetectable at all stages of cortical differentiation *in vitro* (St George-Hyslop et al., 2023). Together, these observations suggest isoform-specific regulatory programs that may confer distinct biological roles, but the developmental and physiological significance of non-canonical isoforms remains poorly understood.

### Expression pattern of CNTNAP2 outside the nervous system

Dysregulation of *CNTNAP2* expression has been reported across multiple tumor types, including childhood neuroblastoma, recurrent oligodendrogliomas, LSCC, HPSCC, and ESCC (Li et al., 2018; Zhang J. et al., 2024). Although these studies did not resolve transcript isoform, they collectively implicate *CNTNAP2* in tumorigenesis and cancer progression.

Notably, recent work has uncovered isoform-specific regulation of *CNTNAP2* in cancer. In OSCC, *CNTNAP2-203*, but not the canonical *CNTNAP2-201*, is predominantly expressed and markedly upregulated in malignant epithelial cells. Elevated *CNTNAP2-203* levels correlate with poorer clinical outcomes in patients (Zhu et al., 2025), highlighting a potential oncogenic role for this short isoform and underscoring the need for isoform-resolved analyses in cancer biology.

Additionally, although *CNTNAP2-201* is most abundantly expressed in the nervous system, low-level expression has been detected in prostate and ovarian tissues (Poliak et al., 1999), suggesting potential functional relevance of CNTNAP2 beyond the CNS.

### Regulation of CNTNAP2 expression

*CNTNAP*2 expression is tightly controlled by multiple transcription factors (TFs), reflecting its complex spatial and temporal regulation in the nervous system. Among the best-characterized regulators are STOX1A, TCF4, FOXP2, and FOXP1 (Figure 3).

STOX1A, a winged-helix TF highly expressed in the brain, acts primarily as a repressor by binding to a regulatory elements within intron 1 of *CNTNAP2* (van Abel et al., 2012). In contrast, TCF4, a basic helix-loop-helix factor enriched in the neocortex and hippocampus, activates *CNTNAP2* transcription through promoter binding. Pathogenic mutations in TCF4 cause PTHS, which shares phenotypic features with *CNTNAP2*-associated disorders, including ID, seizures, and impaired speech (Forrest et al., 2012; Li et al., 2010; Peippo and Ignatius, 2012; Stefansson et al., 2009; Whalen et al., 2012).

FOXP2, a forkhead TF expressed in the cortex, striatum, thalamus, and cerebellum, binds intron 1 of *CNTNAP2* and generally represses its transcription (Adam et al., 2017; Vargha-Khadem et al., 1995, 1998; Watkins et al., 2002). FOXP1, which overlaps with FOXP2 in expression and can form heterodimers to co-regulate target genes (Sin et al., 2015), similarly represses *CNTNAP2* expression (Mendoza and Scharff, 2017). Mutations in both *FOXP1* and *CNTNAP2* have been reported in individuals with ASD and mild-to-moderate ID (O’Roak et al., 2011; Worthey et al., 2013).

Because these TFs bind either to the promoter of the full-length *CNTNAP2-201* or intron 1 which is absent from *CNTNAP2-*203, their regulatory effects likely apply predominantly to *CNTNAP2-201.* These findings also suggest that spatial and temporal differences in transcriptional activators (e.g., TCF4 in cortical layers) and repressors (FOXP1/2 in ganglionic eminences) likely contribute to the region-specific expression patterns of *CNTNAP2-201*.

To date, no TFs have been identified that directly regulate *CNTNAP2-203*. Interestingly, proteomic analyses in *Cntnap2* knockout (KO) mice (lacking the full-length ∼150 kDa Cntnap2/Caspr2 corresponding to *CNTNAP2-201* isoform) revealed increased abundance of a short protein corresponding to the C-terminal region (amino acid 1,224–1,332), which overlaps with CNTNAP2-203 (Chen et al., 2015). This observation raises the possibility of compensatory upregulation of *CNTNAP2-203* upon loss of *CNTNAP2-201*.

In addition to transcriptional control, post-transcriptional mechanisms are likely to fine-tune *CNTNAP2* expression. Given the large size of the *CNTNAP2* transcript, multiple microRNAs (miRNAs) have been predicted to target it. For example, intron 3 contains predicted binding sites for miR-548AR/AQ, and heterozygous deletion involving this region has been reported in individuals with autosomal dominant epilepsy with auditory features, ID, ASD, and schizophrenia, suggesting functional relevance of this regulatory mechanism (Flaherty et al., 2017; Leonardi et al., 2018; Rodenas-Cuadrado et al., 2014, 2016). Additional miRNAs, including miR-324-5p and miR-218-5p, have been implicated in neuronal maturation networks in *Cntnap2* KO mouse model (Sun et al., 2019). However, no miRNAs have yet been experimentally validated as direct regulators of CNTNAP2.

## Context-specific function of CNTNAP2 and its variants

Despite strong associations with diverse human diseases, the biological functions of CNTNAP2 remain incompletely understood. Recent studies, however, have begun to uncover pronounced context-dependent roles for this gene and its variants. The full-length isoform *CNTNAP2-201* (encoding CASPR2/CNTNAP2) is primarily implicated in neurodevelopmental, psychiatric, and autoimmune neurological disorders, where its disruptions contribute to impaired cortical connectivity, interneuron dysfunction, and autoantibody-mediated protein internalization. In addition, multiple proteolytic fragments of CNTNAP2, including the shed extracellular domain and α-secretase/γ-secretase cleaved C-terminal domains, have been shown to regulate CNS functions. By contrast, the short isoform CNTNAP2-203 has emerged as a potential oncogenic factor, being selectively upregulated in OSCC and promoting tumor progression through interactions with EGFR. Collectively, these data highlight the isoform- and context-specific functions of CNTNAP2, spanning essential roles in brain development and synaptic regulation to pathological contributions in cancer biology.

### CNTNAP2 regulates myelination and nodes of Ranvier

In myelinated fibers of the CNS and PNS, oligodendrocytes or Schwann cells wrap axons to form compact myelin, generating insulated internodes separated by short (∼1 μm) nodes of Ranvier (NOR). At NORs, dense clusters of voltage-gated Na^+^ (Nav) channels regenerate action potentials (APs). Flanking paranodes contain terminal myelin loops that form septate-like axo-glial junction with the axolemma, thereby restricting lateral diffusion of channels and electrically isolating the node. Adjacent juxtaparanodes (JXP) are enriched in voltage-gated K^+^ (Kv) channels, which accelerate repolarization and modulate axon excitability (Jensen et al., 2011). This precise molecular compartmentalization underlies efficient saltatory conduction, and the myelination thickness and NOR dimensions determining conduction velocity (Lubetzki et al., 2020).

As a cell-adhesion protein, CASPR2 is concentrated at the axon initial segment and, most prominently, at juxtaparanodes. There, it forms a cis-complex with contactin-2 (CNTN2/TAG-1) and couples to protein 4.1 B, MAGUK scaffolds (PSD93 and PSD95), and ADAM22 via its C-terminal PDZ-binding motif, creating a platform that concentrates and stabilizes Kv1-family channels (Kv1.1/Kv1.2) (Hivert et al., 2016; Ogawa et al., 2008; Poliak and Peles, 2003). In 4.1b-deficient mice, paranodal Caspr2 clustering and Caspr2/Cntn2 localization at JXP are disrupted, demonstrating the essential role of 4.1B in assembling this complex (Cifuentes-Diaz et al., 2011). Functionally, juxtaparanodal Kv1 channels constrain internodal excitability, maintaining the resting membrane potential and shaping AP repolarization during saltatory conduction. Consistent with this model, individuals from CDFE cohorts with homozygous *CNTNAP2* mutations display reduced Kv1.1 localization in hippocampal axons (Strauss et al., 2006), and *Cntnap2*-null mice exhibit diminished Kv1.2 density in cortical myelinated axons, accompanied by enhanced excitatory transmission and increased neurotransmitter release probability (Poliak and Peles, 2003; Scott et al., 2019). Clinically, CASPR2 dysfunction also manifests as neuronal hyperexcitability in autoimmune disorders such as encephalitis, neuromyotonia, and neuropathic pain, in which CASPR2 and CNTN2 serve as autoantigens targeted by autoantibodies (Binks et al., 2018; Habib et al., 2025; Kelly et al., 2024; Patterson et al., 2018).

Beyond JXPs, CASPR2 also contributes to paranodal and nodal organization. At paranodes, CASPR2 and CNTN2 form cis-complexes on the axonal membrane that recruit NF155 from the glial paranodal loops (Gollan et al., 2003). Both CASPR2 and CNTN2 are required for axo-glial junction formation, and disruption of either protein leads to widened nodes and defective nerve conduction (Pinatel et al., 2017). In addition, in *Cntnap2* haploinsufficient mice, overall NOR organization is preserved; however, node length is reduced in a dose-dependent manner, indicating a role for CASPR2 in fine-tuning nodal architecture (Horresh et al., 2010).

CASPR2 further contributes to myelination itself. In the CNS, *Cntnap2*-null mice show transient delays in neocortical myelination and reduced conduction velocity around postnatal week 3, which normalize by week 8, potentially reflecting impaired development of Sox10^+^ oligodendrocytes (Scott et al., 2019). Developmental stage-specific effects on CNS axon caliber have also been reported. Cifuentes-Diaz et al. (2023) observed dose-dependent reductions in axon diameter in the CC at postnatal day 30, but increased caliber in commissural tracts at earlier stages. In the PNS, *Cntnap2* deficiency alters sciatic nerve fiber geometry and increases the g-ratio (indicative of hypomyelination) in a developmentally dependent manner (Cifuentes-Diaz et al., 2023).

Therefore, these findings establish CASPR2 as a principal organizer of juxtaparanodal and paranodal microdomains, stabilizing Kv1 channels and shaping axo-glial junctions. Beyond these canonical roles, CASPR2 modulates node length and exerts tract- and stage-specific effects on axon caliber and myelination in both CNS and PNS.

### CNTNAP2 regulates brain connectivity and excitatory-inhibitory balance

Although CNTNAP2 is best known for its role in organizing axonal microdomains, its high expression during early developmental stages prior to the onset of myelination suggests additional functions in shaping neural circuitry. In mouse cortical neurons, Caspr2 knockdown (KD) reduces dendritic arborization, indicating a role in promoting neuronal connectivity (Gao et al., 2018). Consistently, in human iPSC models, hemizygous deletion of the *CNTNAP2* gene (2.07 Mb, encompassing exons 2–20) disrupts neuronal morphology, characterized by reduced dendritic length and branching, as well as impaired synaptic maturation (Shekhar et al., 2026). These findings are further supported by studies in *Drosophila* lacking *Nrx-IV* (the *CNTNAP2* ortholog) and in humans with *CNTNAP2* mutations, both of which exhibit defects in axonal pathfinding and dendritic morphology, highlighting an evolutionarily conserved role in circuit formation (Banerjee et al., 2010; Strauss et al., 2006).

At the systems level, resting-state functional magnetic resonance imaging (fMRI) in *Cntnap2*-null mice demonstrates reduced local- and long-range functional connectivity, particularly within prefrontal and midline brain regions, affecting heteromodal cortical regions and fronto-posterior components of the default-mode network (Liska and Gozzi, 2016). In contrast, increased functional connectivity has been observed in the primary somatosensory cortex in *Cntnap2*-null mice (Balasco et al., 2022a), indicating region-specific effects on network organization. Human neuroimaging studies further support a role for *CNTNAP2* in regulating brain connectivity. Scott-Van Zeeland et al. (2010) demonstrated that the common variant rs2710102 is associated with altered functional connectivity in the medial prefrontal cortex, characterized by enhanced local connectivity and reduced long-range connectivity. Later structural network analyses by Dennis et al. (2011) also revealed that individuals carrying *CNTNAP2* rs2710102 variant exhibit reduced whole-brain path length and lower eccentricity, suggesting impaired organization of long-range connections. Consistently, more recent fMRI studies have shown that *CNTNAP2* risk variants, including rs2710102, rs2538991, and rs2710126, are associated with reduced cortical thickness and abnormal white matter structure in the cingulate gyrus (Chien et al., 2021; Table 2).

**TABLE 2:** Functions and phenotypes of human *CNTNAP2* variants.

Variant site	Classification	Research method	Phenotype	Disease
rs2710102	Monoallelic	Human magnetic resonance imaging	Enhanced local connectivity; reduced long-range connectivity	Autism spectrum disorder
rs2538991	Monoallelic	Human magnetic resonance imaging	Cortical dysplasia	Autism spectrum disorder
rs2710126	Monoallelic	Human magnetic resonance imaging	Cortical dysplasia	Autism spectrum disorder
D1129H	Biallelic	HEK-293 cells; Mouse hippocampal neurons	Abnormal CNTNAP2 protein transport	Autism spectrum disorder
I1253X	Biallelic	HEK-293 cells	Abnormal subcellular localization	Autism spectrum disorder
R777G	Biallelic	Mouse primary neurons	Excitation/inhibition imbalance	Seizures, language regression, and behavioral abnormalities
N407S N418D G731S T1278I	Biallelic	Mouse medial ganglionic eminence cells	Reduced interneurons	Autism spectrum disorder

Beyond axons, CASPR2 localizes to synapses, dendrites, and spines, where it influences both excitatory and inhibitory transmission (Bakkaloglu et al., 2008; Varea et al., 2015). Ca2^+^-imaging studies show that Caspr2 KD reduces network activity and diminishes AMPA- and NMDA-receptor-mediated excitatory postsynaptic currents, as well as GABA-receptor-mediated inhibitory currents, highlighting a broad role in synaptic signaling. CASPR2 colocalizes with AMPA-receptor subunit GluA1at excitatory synapses, and its loss leads to intracellular GluA1 accumulation and impaired receptor trafficking (Varea et al., 2015). In glutamatergic, inhibitory interneurons, CASPR2 forms a complex with CASK and GluA1 to regulate synaptic GluA1 abundance (Gao et al., 2019).

Above mentioned cellular observations were further supported by electrophysiological analyses using brain slices. In hippocampal slices from *Cntnap2* KO mice, perisomatic evoked inhibitory postsynaptic currents (iPSCs) are reduced, whereas excitatory transmission remains largely unaltered, accompanied by increased spontaneous AP-driven inhibitory activity (Jurgensen and Castillo, 2015). In the prefrontal cortex, KO mice display reduced miniature excitatory postsynaptic current amplitude and decreased excitatory fiber volleys of AP waveform (Kim et al., 2019b; Scott et al., 2019). Similar reductions in both excitatory and inhibitory inputs have been observed in layer II/III pyramidal neurons of the medial prefrontal cortex (mPFC), while *in vivo* recordings reveal immature cortical evoked potentials and neuronal hyperexcitability in *Cntnap2* KO rats (Lazaro et al., 2019; Scott et al., 2020, 2022).

It is important to note that discrepancies remain regarding whether CASPR2 preferentially regulates excitatory or inhibitory transmission. These differences likely arise from variations in experimental conditions, including *in vitro* versus *in vivo* assays, as well as differences in developmental stages. Despite these inconsistencies, a more consistent theme across models is the disruption of the excitatory-inhibitory (E/I) balance. Multiple ASD models involving CNTNAP2 loss-of-function exhibit increased E/I ratios, often driven by enhanced excitatory connectivity. For example, in *Cntnap2* KO mice, the primary somatosensory cortex shows increased expression of excitatory neuronal markers and elevated functional connectivity, while feedforward cortical circuits show reduced inhibition with modest decreases in excitation, resulting in a net increase in the E/I ratio (Antoine et al., 2019; Balasco et al., 2022b). These circuit-level abnormalities are further supported by human genetic studies. ASD-associated *CNTNAP2* variants, such as R777G, disrupt neuronal morphology and function, leading to reduced axonal growth, altered E/I postsynaptic currents, and impaired action potential firing in primary cortical neurons (Lu et al., 2021). Given that *Cntnap2*-deficient rodent models recapitulate key ASD-associated phenotypes, these findings suggest that disruption of E/I balance and large-scale connectivity constitutes a core mechanism underlying *CNTNAP2*-associated neurodevelopmental disorders.

Together, these findings position CNTNAP2 as a key regulator of neuronal connectivity and synaptic balance. By influencing dendritic morphology, synaptic transmission, and network excitability, CNTNAP2 contributes to the establishment and maintenance of functional neural circuits. Disruption of these processes likely underpins the pathophysiology observed in ASD and related NDDs.

### CNTNAP2 regulates neuronal migration and interneuron maturation

Early evidence linking CNTNAP2 to neuronal migration emerged from neuropathological analyses of patients with CDFE carrying *CNTNAP2* mutations. Pathological analyses revealed that affected brains displayed cortical thickening, poorly defined gray-white matter junctions, increased neuronal density in hippocampal and temporal neocortical regions, disorganized neuronal orientation, and abnormal dendritic morphology (Strauss et al., 2006). Consistently, *Cntnap2*-null mice display ectopic neurons in the CC and reduced GABAergic interneurons in the cortex, striatum, and hippocampus (Peñagarikano et al., 2011; Scott et al., 2019), all suggesting a role for CASRP2 in corticogenesis and neuronal differentiation.

GABAergic interneurons, which comprise ∼20% of neocortical neurons, are critical for shaping cortical network activity through temporally precise inhibition over excitatory circuits (Wood et al., 1979). They are commonly classified into Ca2^+^-binding protein parvalbumin (PV), the neuropeptide somatostatin, and the ionotropic serotonin receptor 5HT3a expressing subtypes (Rudy et al., 2011). Multiple studies demonstrate that Cntnap2 deficiency selectively reduces GABAergic interneuron population. In *Cntnap2*-null mice, decreased number of GAD1^+^ interneurons have been reported in the cortex, striatum, and hippocampus at postnatal day 14, in the somatosensory cortex at postnatal day 30, and in the striatum at postnatal day 25 (Lauber et al., 2018; Peñagarikano et al., 2011; Vogt et al., 2018). Similar phenotypes are observed in *Cntnap2*-deficient cortical organoids derived from mouse induced pluripotent stem cells, where specific loss of GABA^+^/GAD1^+^ interneurons is observed and mechanistically linked to downregulation of ventral telencephalic progenitor TFs, including *Dlx2*, *Nkx2.1*, and *Ascl1* (Hali et al., 2020).

Further evidence indicates that autism-associated *CNTNAP2* missense mutations (N407S, N418D, G731S, and T1278I) also impair interneuron development. Vogt et al. (2018) demonstrated that these variants fail to rescue the reduced number of GABAergic interneurons observed in *Cntnap2*-null mice, indicating that they function as loss-of-function mutations the number of GABAergic interneurons was reduced in the cortex of *Cntnap2* KO mice, and transfection with any of these four mutations failed to restore their numbers (Table 2).

Beyond interneuron abundance, CASPR2 also modulates interneuron function. In the mPFC of *Cntnap2*-null mice, PV^+^ interneurons exhibit markedly reduced activity during social behaviors. Remarkably, selective activation of PV^+^ interneuron or inhibition of excitatory neurons restored the E/I balance and rescued social deficits in these mice (Selimbeyoglu et al., 2017). These findings highlight CNTNAP2 as a critical regulator of interneuron development and function, whose disruption alters both cellular composition and circuit activities.

### Emerging roles of CNTNAP2 protein fragments

Historically, most functional studies of CNTNAP2 have focused on the full-length protein, typically through KO, KD, or mutations affecting regions unique to the *CNTNAP2-201* isoform. However, recent work has begun to uncover an additional layer of complexity involving proteolytic processing of CNTNAP2 and the generation of bioactive protein fragments. These include the shed extracellular ectodomain (CNTNAP2-ecto), the γ-secretase-derived intracellular domain (CICD), and the α-secretase produced C-terminal fragments C79. Given the unusually large size of CNTNAP2 and its complicated extracellular, transmembrane, and intracellular domains, it is likely that additional functionally relevant fragments remain to be identified.

Full-length CNTNAP2 undergoes neuronal activity-dependent ectodomain shedding mediated by the matrix metallopeptidase 9 (MMP9). The released ectodomain is detectable in human and mouse cerebrospinal fluid (CSF), neuronal culture media, and soluble cortical fractions of mouse brain. Notably, CNTNAP2-ecto levels are significantly reduced in the CSF of individuals with ASD, suggesting disease relevance. Functionally, CNTNAP2-ecto binds plasma membrane Ca2^+^ ATPase2 (PMCA2), enhancing Ca2^+^ extrusion and modulating neuronal network dynamics in a PMCA2-dependent manner (Martín-de-Saavedra et al., 2022). These findings reveal that ectodomain shedding represents not merely a degradation pathway, but an active signaling mechanism that couples neuronal activity to calcium homeostasis.

**FIGURE 4:**
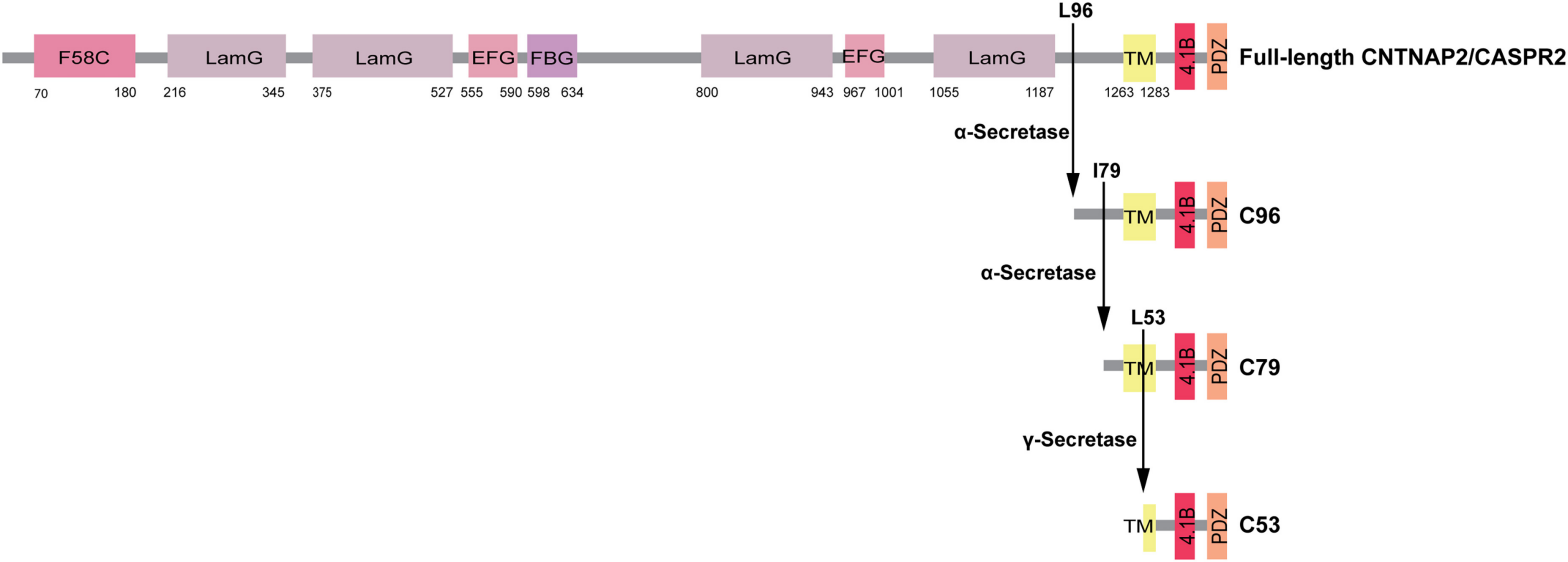
Schematic illustration of the sequential proteolytic processing of full-length contactin-associated protein-like 2 (CNTNAP2). Full-length CNTNAP2 undergoes stepwise proteolytic cleavage initiaed by ADAM10/17-dependent α-secretase at extracellular sites I79 and L96. ADAM10 primarily cleaves at 179 to produce the predominant C-terminal fragments C79, and ADAM17 preferably cleaves at L96 to produce the C-terminal fragments C96. C96 can be further processed by α-secretase to yield C79. Subsequently, γ-secretase cleaves C79 at L53 within the transmembrane domains to generate the C-terminal fragments C53. Distinct structural domains of full-length CNTNAP2 protein are depicted as colored rectangles, as indicated in the legend. Domain abbreviations are as follows: F58C, discoidin-like domain; LamG, laminin G-like domain; EFG, epidermal growth factor-like domain; FBG, fibrinogen-like domain; TM, transmembrane domain; 4.1B, protein 4.1B-binding motif; PDZ, PDZ-binding motif.

Subsequent studies have demonstrated that full-length CNTNAP2 undergoes sequential proteolytic cleavage, first by ADAM10/17-dependent α-secretase at extracellular sites (I79 and L96, corresponding to residues 1,254 and 1,237 of CASPR2, respectively) and then by presenilin-dependent γ-secretase at residue L53 (corresponding to residue 1,280 of CASPR2). This process generates three major C-terminal fragments-C96, C79, and C53-corresponding to residues 1,237–1,332, 1,254–1,332, and 1,280–1,332 of CASPR2 (Figures 1, 4). Among them, the CICD fragment has been shown to ameliorate autism-related behavioral phenotypes in *Cntnap2* KO mice by facilitating the nuclear translocation of the scaffolding protein CASK and regulating the expression of neurodevelopmental gene *Necdin*. Conversely, ASD-associated mutations in CNTNAP2 impair α-cleavage, reducing C79 generation and leading to ASD-like behavior abnormalities in *Cntnap*^–/*I*1254*T*^ knock-in mice (Zhang Q. et al., 2024). Remarkably, exogenous expression of C79 rescues these behavioral phenotypes, underscoring its functional significance (Zhang Q. et al., 2024).

Collectively, these findings indicate that proteolytic processing of CNTNAP2 represents additional implications in human diseases. Future work is likely to uncover further fragments and mechanisms, expanding the functional repertoire of CNTNAP2 beyond its canonical role as a full-length adhesion molecule.

### CNTNAP2-203 regulates tumor oncogenesis

Although the functional roles of *CNTNAP2-203* (the short isoform of *CNTNAP2*) have not been characterized in the nervous system, emerging evidence implicates this isoform in tumorigenesis. In OSCC, *CNTNAP2-203*, but not the canonical *CNTNAP2-201*, is selectively upregulated in tumor epithelial cells, and its elevated expression correlates with poor clinical prognosis (Zhu et al., 2025). Functional studies further demonstrate that CNTNAP2-203 accelerates tumor cell proliferation *in vitro* and promotes tumor growth and progression in xenograft mouse models.

Mechanistically, co-immunoprecipitation and site-directed mutagenesis studies demonstrate that CNTNAP2-203 directly interacts with epidermal growth factor receptor (EGFR), leading to amplification of EGFR signaling and activation of downstream E2F1 pathways. Enhanced CNTNAP2-203-EGFR-E2F1 signaling promotes tumorigenic behaviors, including increased cell proliferation, migration, and survival. Structural analyses provide further insight into this interaction. CNTNAP2-203 shares an identical intracellular domain with CICD, but differs in membrane topology: CNTNAP2-203 retains a full transmembrane domain and a short extracellular region, whereas CICD lacks most of the transmembrane segment. Notably, only CNTNAP2-203, and not CICD, binds to and modulates EGFR, indicating that the transmembrane domain is essential for EGFR regulation (Zhu et al., 2025).

Together with emerging evidence on proteolytic fragments of CNTNAP2, these findings further highlight a previously unrecognized, non-canonical role of CNTNAP2 isoforms. More broadly, they underscore the importance of isoform-resolved functional analyses to elucidate the context-dependent roles of *CNTNAP2* across neurological and oncological diseases.

## Translational implications of CNTNAP2

Current research on CASPR2 in disease contexts has yielded substantial mechanistic insights. However, the translational application of CNTNAP2-related findings remains at an early stage. In autoimmune encephalitis, evidence supports the efficacy of combination immunotherapy based on CNTNAP2 antibody detection. For example, in patients positive for anti-LGI1/CASPR2 antibodies, treatment with intravenous immunoglobulin or methylprednisolone significantly improves modified Rankin Scale scores compared with untreated individuals or those receiving monotherapy (Rittel et al., 2025). Although most patients respond favorably to immunotherapy, CASPR2-associated encephalitis is characterized by a relatively high relapse rate (Brand et al., 2026). In addition, patients with elevated anti-LGI1/CASPR2 antibody levels exhibit an increased risk of relapse; however, this risk can be significantly reduced by long-term corticosteroid therapy or repeated rituximab administration.

In oncology, CNTNAP2-203 has been proposed as a molecular subtyping biomarker in OSCC, with potentially utility in guiding patient selection for EGFR-targeted therapies such as gefitinib (Zhu et al., 2025). However, current evidence is still largely limited to cellular and preclinical models, and its clinical value requires validation in large, well-controlled clinical research.

In neurodevelopmental disorders, particularly ASD, human iPSC-derived brain organoid models with *CNTNAP2* deficiency have provided important mechanistic insights, including accelerated cell cycle, disorganized ventricular zone structure, imbalanced GABAergic and glutamatergic neuron differentiation, and activation of the AKT-mTOR pathway (Chalkiadaki et al., 2025; Jang et al., 2025; Kim et al., 2019a). Despite these advances, translation into clinically usable interventions remains unresolved.

Future efforts should prioritize the development of precision diagnostic driven by molecular biomarkers, alongside the development of coordinated, multi-dimensional therapeutic strategies. In autoimmune encephalitis, antibody subtype-guided immunotherapy and monitoring of voltage-gated potassium channel (VGKC) complex antibody titers may improve patient stratification and enable early detection of relapse. In oncology, isoform-specific biomarkers such as CNTNAP2-203 may facilitate more precise patient selection for targeted therapies. In neurodevelopmental diseases, human iPSC-based platforms offer scalable systems for drug screening and therapeutic discovery. Collectively, disease-specific translational strategies informed by underlying biological mechanisms will be essential for advancing CNTNAP2-targeted precision medicine.

## Conclusion and perspectives

Over the past two decades, CNTNAP2 has emerged as a pleiotropic and context-dependent regulator implicated in a wide spectrum of human diseases, ranging from neurodevelopmental and psychiatric disorders to autoimmune encephalitis and multiple malignancies. Early studies focused primarily on the full-length isoform *CNTNAP2-201* (encoding CNTNAP2 or CASPR2), establishing its indispensable roles in neuronal function. CASPR2 organizes juxtaparanodal domains, stabilizes Kv1 channels, fine-tunes excitatory-inhibitory balance, regulates neuronal migration and interneuron maturation, and maintains neuronal network connectivity. These foundational discoveries positioned CNTNAP2 as a key molecule in axon-glia interactions, synaptic regulation, and cortical circuit dynamics.

More recent findings are reshaping this classical view by uncovering isoform-specific and proteolytic fragment-dependent functions of CNTNAP2. Proteolytic processing by MMP9, α-secretases, and γ-secretases generates functionally distinct protein fragments, including shed ectodomains that bind PMCA2 to regulate calcium homeostasis and intracellular domains that interact with CASK to modulate neuronal gene expression. In parallel, discoveries in oncology have identified the short isoform *CNTNAP2-203* as an unexpected oncogenic driver in OSCC, where its selective upregulation amplifies EGFR-E2F1 signaling and promotes tumor progression. Collectively, these advances reposition CNTNAP2 not merely as a neuronal adhesion molecule, but as a dynamic signaling platform diversified through alternative splicing and regulated proteolytic cleavage.

Despite these advances, several key questions remain. First, the regulation, spatial distribution, and biological functions of non-canonical CNTNAP2 isoforms remain largely unexplored. Systematic, isoform-resolved profiling across tissues, developmental stages, and disease contexts will be essential to define their biological niches. Second, although *Cntnap2*-null mouse models have provided a valuable tool for studying the gene function and neurodevelopmental mechanisms, their limitations must be carefully considered. Significant interspecies differences in cortical development, neural network architecture, and synaptic plasticity limit the ability of rodent models to fully interpret the complex clinical phenotypes observed in humans. Likewise, traditional *in vitro* systems, including primary neuron cultures and immortalized cell lines, lack the multicellular microenvironment and three-dimensional architecture required to model higher-order network-level pathology. Third, although accumulating evidence implicates CNTNAP2 proteolytic fragments in neuronal signaling and ASD pathogenesis, their downstream pathways and regulatory mechanisms remain incompletely understood. Fourth, the emerging oncogenic role of CNTNAP2-203 raises important questions about whether and how other isoforms or proteolytic fragments contribute to tumorigenesis in diverse contexts. Finally, advancing CNTNAP2-focuses on translational research will be essential to realize its therapeutic potential in human disease.

In summary, CNTNAP2 represents a multifunctional, context-dependent regulator across neurology and oncology. Elucidating its multilayered regulation and isoform-specific functions will be critical for understanding both its physiological roles and its contributions to human diseases. Such knowledge may ultimately enable the development of isoform- or fragment-specific therapeutic strategies targeting CNTNAP2-driven neurological disorders and cancers.

## Footnotes

1
https://www.hgmd.cf.ac.uk/ac/index.php

2
http://stemcell.libd.org/scb
